# Perception of Fish Sentience, Welfare and Humane Slaughter by Highly Educated Citizens of Bogotá, Colombia and Curitiba, Brazil

**DOI:** 10.1371/journal.pone.0168197

**Published:** 2017-01-09

**Authors:** Daniel Santiago Rucinque, Ana Paula Oliveira Souza, Carla Forte Maiolino Molento

**Affiliations:** Animal Welfare Laboratory, Federal University of Paraná, Curitiba, Paraná, Brazil; Universidade do Porto Instituto de Biologia Molecular e Celular, PORTUGAL

## Abstract

Discussions on farm animal welfare have become frequent, especially in developed countries. The aim of this research was to study the perception of fish sentience, welfare and slaughter by highly educated citizens from Bogotá, Colombia, and Curitiba, Brazil. An online survey with 12 questions presented as open-ended, multiple choice and 5-point Likert-type scale formats was available to respondents. Answers from 395 participants in Bogotá and 387 in Curitiba were analyzed, and results are presented in the order Bogotá followed by Curitiba. The percentage of participants who perceived fish as sentient animals was 79.7% and 71.8%. The classification of sentience perception among taxonomic groups seems in accordance with the phylogenetic proximity to humans, suggesting participants were more likely to perceive sentience in mammals than in other animals. The descending order related to the highest perception of fish suffering in different scenarios was fishing with hook and line (75.6%, 70.6%); municipal live fish fair (68.7%—only in Curitiba); fish-and-pay ponds (59.7%, 54.4%); fish kept as laboratory animals (58.0, 48.1%); fish farming (35.7, 36.8%); fish in pet stores (35.5%, 26.1%); production of ornamental fish (19.3%, 21.8%); fish in aquarium exhibits (18.8%,16.9%); and fish kept as pets (12.4%,12.3%). Lack of knowledge about the conditions of capture, handling, transport and sale of ornamental fish may justify the perception of low level of suffering in the last scenarios. Regarding humane slaughter, 57.0% and 55.0% of respondents were unaware of the issue. After reflection induced by the questionnaire, 76.0% and 72% of participants believed that fish should be included in humane slaughter regulations. This study presents original data suggesting that respondents from Bogotá and Curitiba consider fish as sentient beings. The perception of suffering in specific scenarios challenges common activities. Recognition of suffering also endorses humane slaughter regulations to reduce pain in a large number of individuals of fish slaughtered annually for human consumption in Colombia and Brazil.

## Introduction

Debates on farm animal welfare have become frequent, especially in developed countries. Citizens of developed countries have put pressure for higher animal welfare standards for sentient animals [1]. Broom defined a sentient animal as one that has some ability to: (i) evaluate the actions of others in relation to itself and third parties; (ii) remember some of its own actions and their consequences; (iii) assess risks and benefits; (iv) have some feelings; and (v) have some degree of awareness [2]. In addition, Webster defined that sentient animals are those that experience emotions associated with pleasure and suffering [3].

In relation to fish, research has revealed that teleost fish can feel pain and that they present memory and consciousness [4–10]. Furthermore, fish are known to fall for optical illusions, which suggest that they not only take in visual information but complex processing also occur at the level of perceptual organization [11]. Unlike mammals, many fish are capable of detecting and creating electric currents in the water, allowing them to find prey, navigate in their environment and communicate with their fellows [12]. Learning association between sound and food occurred after 14 trials in rainbow fish [13], while in rats it took about 40 trials [14]. Fish have excellent long-term memories, develop complex traditions, show signs of Machiavellian intelligence, cooperate with and recognise one another and are even capable of tool use [12,15,16]. Fish have neuroanatomical, behavioural and physiological attributes that characterize conscious cognition or motivational affective states, thus there seems to be compelling evidence to suggest that fish can suffer [7], even though a small number of papers argue against fish sentience [17]. Thus, on balance, scientific evidence seems to suggest that fish are sentient beings.

Concerns about fish sentience and capacity to suffer have contributed to improve animal handling and promoted practices that may increase animal welfare and avoid suffering states. Recently, the World Organization for Animal Health, through the Aquatic Animal Code, set recommendations for the welfare of farmed fish, mainly in transport, stunning and killing for human consumption [18]. This is particularly relevant since fish are the most consumed animal and are the second most used in scientific research worldwide [12,19].

In Norwegian and Swedish humane slaughter regulations, stunning of farmed fish is mandatory [20,21]. Stunning is applied to animals to induce unconsciousness and insensibility to pain stimuli for sufficient duration to secure that an animal remains unconscious until it is killed [22]. Killing methods such as asphyxia on ice, evisceration and exsanguination cause a high degree of suffering in conscious animals [23]. In Latin American countries, like Brazil and Colombia, these methods are commonly used without previous stunning, as fish are not included in the humane slaughter regulations [24,25].

Public opinion is an important factor when discussing new regulations to improve fish welfare in different scenarios [26]. However, it seems that people are poorly informed about livestock production systems. For example, in Brazil, Bonamigo et al observed that respondents did not know broiler chicken production [27], and Pedrazzani et al observed that respondents had never heard about humane slaughter of animals [28].

Considering the increasing demand for products of animal origin, it is necessary to study the perception of society in relation to the quality of life of animals, because social concern dictates the need for animal welfare standards and regulations [29]. From such knowledge, changes in production systems and regulations may be proposed to improve fish welfare. Thus, the aim of this research was to study the perception of fish sentience, welfare and slaughter by highly educated citizens from Bogotá, Colombia, and Curitiba, Brazil.

## Materials and Methods

An online survey containing 12 questions was made available through the Survio^®^ platform to the citizens of Bogotá (BOG), Capital District, Colombia and Curitiba (CWB), Paraná, Brazil, in the language of each country (Table 1). Questions were presented as open-ended, multiple choice and 5-point Likert-type scale formats. Additionally, participants were asked demographics questions, such as gender, age and education. The minimum sampling size of 384 respondents in each city was calculated based on Schaeffer et al. considering a maximum error of 5.0% for a population of over one million people; a 95.0% confidence interval and a maximum uncertainty principle of 50.0% [30]. The survey was distributed by e-mail and social networks. In addition, coordinators of higher education programs in both cities were contacted by e-mail to share the survey link amongst professors and students. A total of 20 universities in BOG and 5 in CWB were contacted. Responses were collected from August 2014 to April 2015. The study was approved by the Human Research Ethics Committee of the Federal University of Paraná (SCS/UFPR), protocol 814–835 2014.

**Table 1:** Non-demographic questions (Q) included in the online survey about the perception of fish sentience, welfare and slaughter in Bogotá, Capital District, Colombia and Curitiba, Paraná, Brazil; available from August 2014 to April 2015.

Questions	Content	Options of answers
Q1	Do you believe fish can feel emotions such as fear and joy, and also suffer? Why?	1 certainly not; 2 no; 3 undecided or I do not have an opinion on this; 4 yes; 5 certainly yes.
Q2	On a scale from 1 to 5, classify the ability of each animal to feel emotions: pigeon, butterfly, human baby, rat, dog, chicken, fish, sheep, cattle, cockroach and wolf.	1; 2; 3; 4; 5; I do not know. 1 means do not feel emotions; 5 means certainly feel emotions; intermediate values are equivalent of a growing capacity to feel emotions.
Q3	Do you believe that removing fish from the water causes suffering to them?	1 certainly not; 2 no; 3 undecided or I do not have an opinion on this; 4 yes; 5 certainly yes.
Q4	Is catch-and-release angling acceptable? Why?	1 certainly not; 2 no; 3 undecided or I do not have an opinion on this; 4 yes; 5 certainly yes.
Q5	Have you ever practiced angling?	Yes, almost every day; yes, 1–3 times a week; yes, 1–3 times a month; yes, a few times a year; yes, only once; no, never.
Q6	On a scale from 1 to 5, in which 1 = none and 5 = maximum, how much pain do you believe fish can experience?	1; 2; 3; 4; 5.
Q7	What is the level of fish suffering in the following scenarios? Fish with hook and line; municipal live fish fair (*C); fish-and-pay ponds; fish kept as laboratory animals; fish farming; fish in pet stores; production of ornamental fish; fish in aquarium exhibits and fish kept as pets.	1 none; 2 a little; 3 moderate; 4 a lot; I do not know.
Q8	Which methods of fish slaughter have you ever seen?	Asphyxia; gutting; decapitation; percussion; death in dry plastic bags (*C); none; others:
Q9	Do you believe that the methods of slaughter you indicated in the previous question cause suffering to the fish?	1 certainly not; 2 no; 3 undecided or I do not have an opinion on this; 4 yes; 5 certainly yes.
Q10	Do you think suffering affects fish meat quality?	1 certainly not; 2 no; 3 undecided or I do not have an opinion on this; 4 yes; 5 certainly yes.
Q11	Have you ever heard of humane slaughter?	Yes; no
Q12	Do you believe that fish should be included in the humane slaughter regulation, as other animals used for food production? Why?	1 certainly not; 2 no; 3 undecided or I do not have an opinion on this; 4 yes; 5 certainly yes.

*C only presented to respondents in Curitiba

Questions 7 and 8 were not introduced to the respondents from BOG because in Colombia there is no similar scenario of a fair where fish are sold alive and are taken home by consumers without water supply.

From a total of 903 respondents, 782 answers were evaluated, 395 from BOG and 387 from CWB, data in S1 Dataset. One hundred and twenty-one respondents were from cities other than BOG or CWB and their responses were not included in our analysis. Considering the demographic variables within the age group, the category "56 years-old or more" presented very few participants, as can be seen in Table 2. Thus, during data analysis, this category was combined with "46–55 years-old". Answers were not compared according to the education level of participants since 95.1% (744/782) of them declared themselves as having higher education (Table 2); this is related to our respondent-request strategy.

**Table 2:** Demographic data of 395 respondents in Bogotá and 387 in Curitiba, from a survey concerning the perception of fish sentience, welfare and slaughter; August 2014 to April 2015.

Variable	Categories	Number of respondents (%)	
		Bogotá	Curitiba
Sex	Male	153 (38.7)	143 (36.9)
	Female	242 (61.3)	244 (63.5)
Age (years)	18–25	202 (51.1)	190 (49.1)
	26–35	110 (27.8)	106 (27.4)
	36–45	47 (11.9)	44 (11.4)
	46–55	27 (6.8)	34 (8.8)
	56 or more	9 (2.3)	13 (3.4)
Education	Primary (incomplete)	1 (0.3)	1 (0.3)
	Primary (complete)	1 (0.3)	1 (0.3)
	Secondary (complete)	26 (6.6)^1^	8 (2.1)^2^
	Higher (incomplete graduation*)	157 (39.7)	176 (45.5)
	Higher (complete graduation)	88 (22.3)	85 (22.0)
	Higher (post graduation)	122 (30.9)	116 (30.0)

Different superscripts report statistical difference (P<0.05) between cities using Chi-square test.

* Graduation in progress or not concluded

Data were analyzed by city, including analysis according to gender and age groups. Descriptive statistics were applied and the Mann-Whitney test was used for comparison in pairs between BOG and CWB and between male and female respondents. The comparison within the four age groups was performed using the Kruskal-Wallis test, followed by Dunn's test when significant difference was found. Both tests were applied in Q1, Q2, Q3, Q4, Q6, Q7, Q9, Q10 and Q12. In Q5, Q8 and Q11 we used the Chi-square test. In addition, demographic data were compared between cities through Chi-square test. The significance level was set at P<0.05. Data was analyzed using Minitab software, version 17.

## Results

### Demographic data

Results regarding respondent gender and educational level can be seen in Table 2. Most respondents were women, 18 to 25 years-old and presented incomplete higher education.

### Fish sentience

#### Sentience perception according to geographic origin

**Fig 1:**
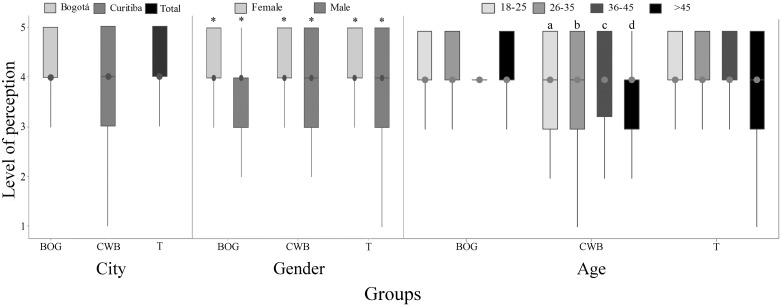
Perception of fish sentience (Q1) by 782 citizens of Bogotá and Curitiba, 2014/2015. Data for city, gender and age; 1 = certainly not and 5 = certainly yes; (BOG) represents data from Bogotá, (CWB) data from Curitiba and (T) the total data; the asterisk indicates gender difference (P<0.05, Mann-Whitney); letters indicate differences between age groups (P<0.05, Kruskal-Wallis and Dunn Test).

In relation to the perception of fish sentience, 79.7% of respondents from BOG and 71.8% from CWB, believed that fish are able to feel emotions (P = 0.6195; Fig 1). Some of the reasons cited by these respondents were: “fish have a nervous system and sensory receptors like other vertebrates”, woman from BOG, 18–25 years old and secondary education; “fish have a complex nervous system that allows them to feel suffering, pain and fear”, man from BOG, 26–35 years old and higher education; “I believe fish feel fear, suffering and pain, but I am not sure about joy”, woman from CWB, 18–25 years old and secondary education; “As any other animal, fish are able to perceive what is happening around them and to react to stimuli”, woman from CWB, 18–25 years old and higher education. Most respondents (75.8%) considered fish as sentient beings.

Few respondents, 8.4% in BOG and 10.9% in CWB, believed fish are unable to feel emotions. Some reasons cited by them were: “animals act by instinct”, man from BOG, over 45 years old and higher education; “fish react to external stimuli, but they have no awareness to feel emotions”, man from BOG, 18–25 years old and higher education; “fish are irrational and emotion presupposes rationality not to be confused with instinct”, man from CWB, over 45 years old and higher education; “because fish are not as developed as mammals”, woman from CWB, 18–26 years old and higher education.

#### Sentience perception according to gender and age of respondent

When data were compared by gender, women gave significantly higher scores to fish sentience in both cities (P<0.01; Fig 1). Comparisons between age groups showed lower perception of fish sentience in respondents older than 45 years old in CWB (P = 0.045; Fig 1). The perceptions of respondents from BOG presented less variance in comparison to respondents from CWB (Fig 1).

#### Sentience perception according to animal species

**Fig 2:**
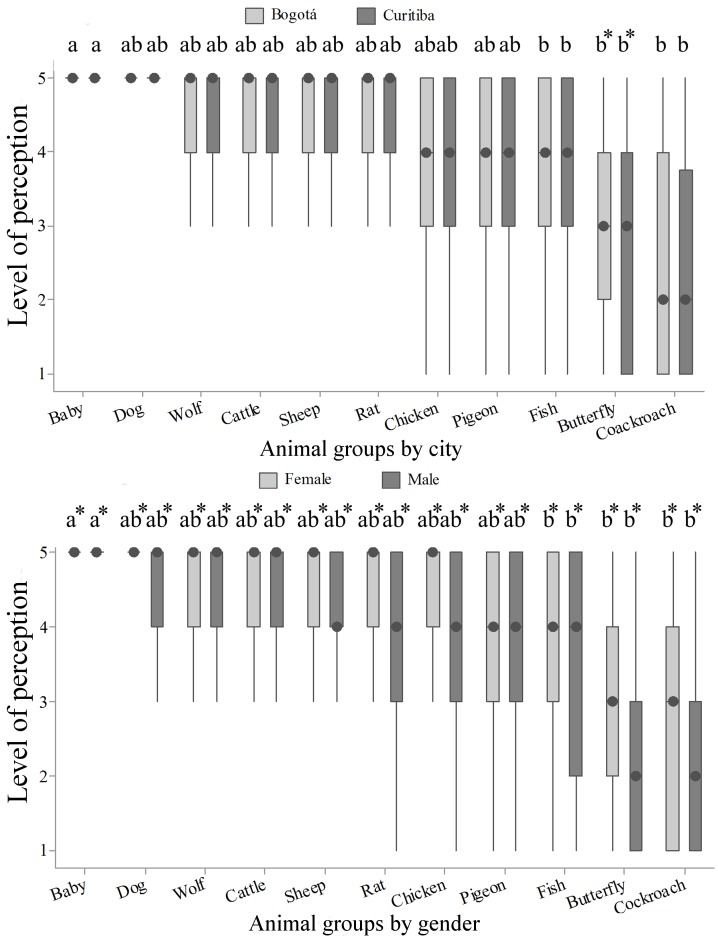
Perception of animal sentience (Q2) by 782 citizens of Bogotá and Curitiba, 2014/2015. Data for city and gender; 1 = no sentience and 5 = maximum sentience; the asterisk indicates both city and gender difference (P<0.05, Mann-Whitney); letters indicate differences between animals (P<0.05, Kruskal-Wallis and Dunn Test).

The respondents rated lower levels of sentience to animals that are more phylogenetically distant from humans (Fig 2). The percentage of respondents who scored "I do not know" for each taxonomic group, presenting Bogotá results followed by Curitiba’s, was: human baby (1.3 and 1.5%); dog (0.8 and 2.1%); wolf (2.8 and 3.9%); cattle (2.0 and 3.9%); sheep (3.3 and 4.7%); rat (2,0 and 5,7%); chicken (2.3 and 6.4%); pigeon (3.0 and 7.0%); fish (4.5 and 8.8%); butterfly (11.9 and 15.5%) and cockroach (13.7 and 15.2%).

Comparison among humans, mammals and birds showed no significant differences, which is probably related to the high variance of the attributed scores. Comparing perception of fish sentience with other taxonomic groups, significant higher scores of sentience were assigned to human baby (P<0.01; Fig 2).

**Fig 3:**
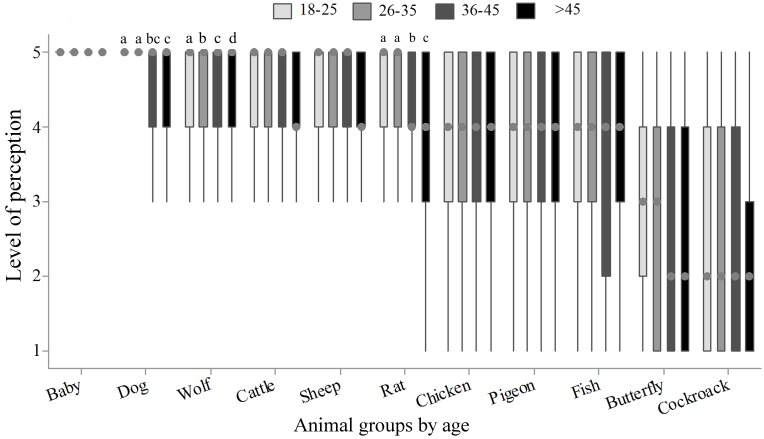
Perception of animal sentience (Q2) data for age, by 782 citizens of Bogotá and Curitiba, 2014/2015. 1 = no sentience and 5 = maximum sentience; letters indicate differences between age groups (P<0.05, Kruskal-Wallis and Dunn Test).

When the perception of sentience within species was compared by gender, women presented higher scores in all groups of animals (P<0.01, Fig 2). Classification and comparison of scores according to age is observed in Fig 3. Scores given to dog, wolf and rat were different within age groups (P<0.05). Despite the fact that wolf scores presented the same median and variance, Dunn's test allowed classification of scores by average rank. Respondents of age group higher than 45 years old tended to assign lower scores to the cattle (P = 0.06), and assigned lower scores for dog, wolf and rat.

### Fish suffering

#### Perceptions about fishing

**Fig 4:**
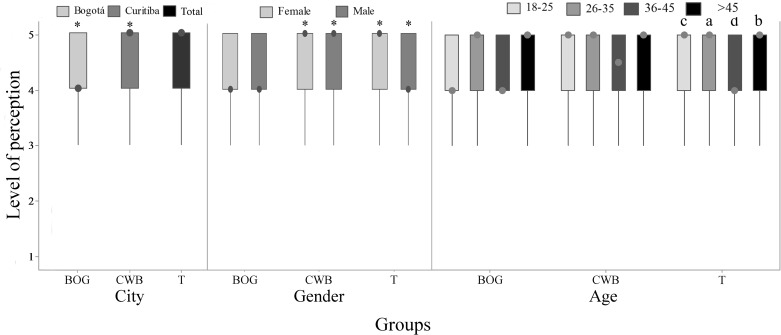
Perception of fish suffering out of water (Q3) by 782 citizens of Bogotá and Curitiba, 2014/2015. Data for city, gender and age; 1 = certainly not and 5 = certainly yes; (BOG) represents data from Bogotá, (CWB) data from Curitiba and (T) the total data; the asterisk indicates city and gender difference (P<0.05, Mann-Whitney); letters indicate differences between age groups (P<0.05, Kruskal-Wallis and Dunn Test).

**Fig 5:**
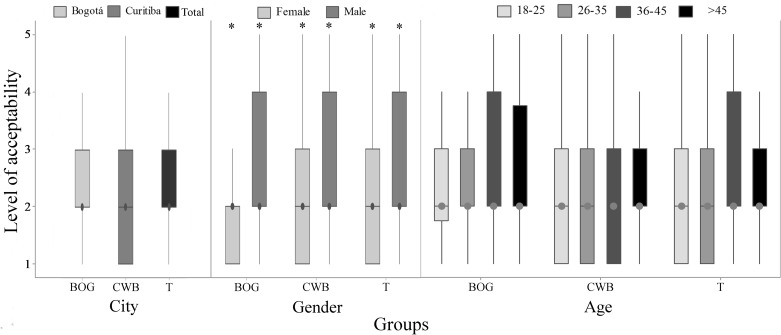
Acceptability of catch-and-release angling (Q4) by 782 citizens of Bogotá and Curitiba, 2014/2015. Data for city, gender and age; 1 = certainly not and 5 = certainly yes; (BOG) represents data from Bogotá, (CWB) data from Curitiba and (T) the total data; the asterisk indicates gender difference (P<0.05, Mann-Whitney).

Most respondents in BOG (93.7%) and CWB (92.8%) declared they recognize that fish suffer when they are removed from the water (P = 0.005) and greater perception of fish sentience was found among citizens of CWB (Fig 4). In addition, women have recognized such suffering more than men in CWB, resulting in difference between gender in general data (P<0.05; Fig 4). When perception of fish suffering was compared per age group, the group of over 45 years old showed lower perception of fish suffering out of the water (P = 0.038) (Fig 4). Few respondents, 19.5% from BOG and 19.9% from CWB, classified catch-and-release angling as an acceptable practice (P = 0.6559; Fig 5), and there were no differences of acceptability when comparison was made per age groups (P>0.05; Fig 5). Some reasons cited for accepting this practice were: "since it is a practice in which fish does not die, I think that it may be an acceptable practice" (man from BOG, 36–45 years old and higher education); “because fish will survive after being released” (woman from CWB, 18–25 years old and higher education).

Most respondents from BOG, 67.3%, and CWB, 64.3%, did not accept catch-and-release angling as a recreational practice (Fig 5), being women more opponent than men in both cities (P<0.01; Fig 5). Common causes for rejecting this practice included the capacity of fish to suffer when removed from the water and the unnecessary injuries caused to animals for human entertainment. As example, one respondent declared “even if fish are slaughtered, there is no justification to catch them only for fun”, man from BOG, 25–35 years old and higher graduation; or “it causes unnecessary suffering of animal in my point of view”, woman from CWB, 36–45 years old and higher education.

Fewer respondents from BOG have already practiced angling when compared to CWB (11.1% and 31.3%, P<0.01), and angling is predominantly performed by men in BOG and CWB (P<0.01). About 63.2% of women declared they had never practiced angling in BOG, against 36.6% of men; and 30.3% of women in CWB, against 12.6% of men. Total of respondents that had never practiced angling were 54.2% in BOG and 22.7% in CWB (P<0.01).

#### Perception of fish pain

**Fig 6:**
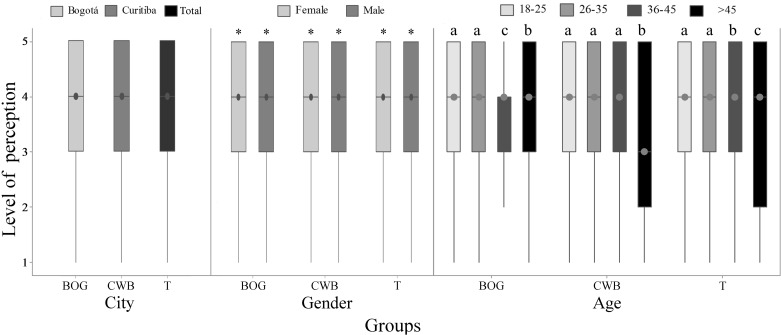
Perception of fish pain (Q6) by 782 citizens of Bogotá and Curitiba, 2014/2015. Data for city, gender and age; 1 = none and 5 = maximum; (BOG) represents data from Bogotá, (CWB) data from Curitiba and (T) the total data; the asterisk indicates gender difference (P<0.05, Mann-Whitney); letters indicate age difference (P<0.05, Kruskal-Wallis and Dunn Test).

Median score for perception of fish pain was 4.0 (1.0–5.0) in both cities (P = 0.1267; Fig 6). In general, few respondents believed fish do not feel pain, 2.3% in BOG and 5.4% in CWB. Gender and age also influenced this result, since women and young people gave higher scores (P<0.01; Fig 6).

#### Perception of fish suffering in different scenarios

**Fig 7:**
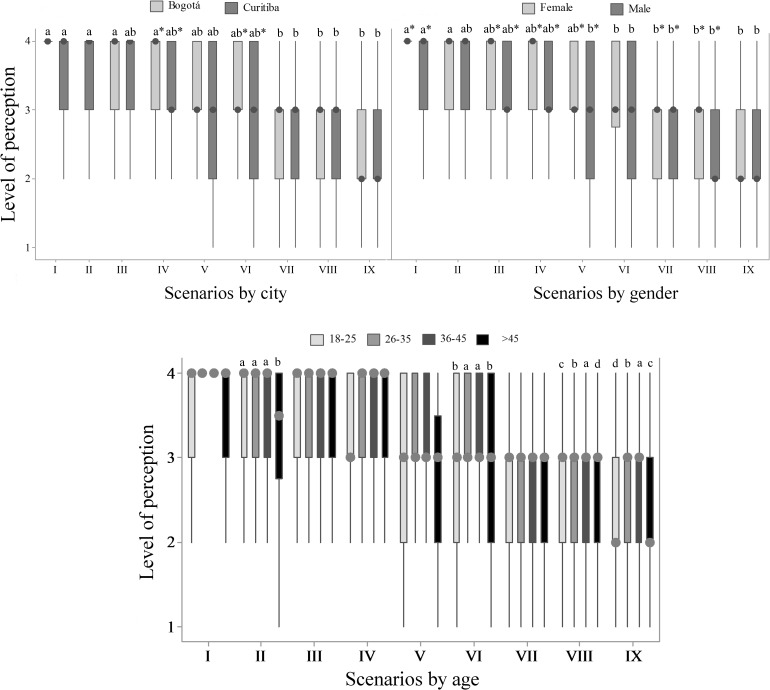
Perceived level of suffering in different scenarios (Q7) by 782 citizens of Bogotá and Curitiba, 2014/2015. Data for city, gender and age; 1 = none and 4 = a lot; **(I)** Fishing with hook and line, **(II)** Municipal live fish fair, **(III)** Fish-and pay ponds, **(IV)** Fish kept as laboratory animals, **(V)** Fish farming, **(VI)** Fish in pet stores, **(VII)** Production of ornamental fish, **(VIII)** Fish in aquarium exhibits, **(IX)** Fish kept as pets; the asterisk indicates city and gender difference (P<0.05, Mann-Whitney); letters indicate differences between scenarios and age groups (P<0.05, Kruskal-Wallis and Dunn Test).

Perception of fish suffering was different according to each scenario, as observed on Fig 7. Descending order of maximum perception of fish suffering, presenting Bogotá results followed by Curitiba’s, was fishing with hook and line (75.6 and 70.6%); municipal live fish fair (68.7% only CWB); fish-and-pay pond (59.7 and 54.4%); fish kept as laboratory animals (58.0 and 48.1%); fish farming (35.7 and 36.8%); fish in pet stores (35.5 and 26.1%); production of ornamental fish (19.3 and 21.8%); fish in aquarium exhibits (18.8 and 16.9%) and fish kept as pets (12.4 and 12.3%). Two scenarios differed between BOG and CWB, fish kept as laboratory animals and in pet stores (P<0.05). These scenarios presented great variance in CWB (Fig 7), also observed in other questions, while respondents from BOG attributed significantly higher scores.

Similar results were observed between women and men regarding municipal live fish fair. Women presented a tendency of higher perception of fish suffering in pet stores (P = 0.055) and significant higher scores in all other scenarios (P<0.05; Fig 7). Comparison between age groups also differed in four scenarios (P<0.05, Fig 7), and in some cases, e.g. fish in pet stores and kept as pet, young age groups did not give the higher scores.

About 61.2% of respondents from CWB answered that fish suffer during live fair. Maximum perception of fish suffering on fish-and-pay pond scenario (59.7% BOG and 54.4% CWB) was lower than fishing with hook and line (75.6% BOG and 70.6% CWB). Fish kept as laboratory animals were also perceived by respondents as involved in animal suffering (58.9% BOG and 48.1% CWB; P<0.05).

Fish farming (P>0.05; BOG 41.3%, CWB 31.5%), fish in pet stores (P<0.01; 41.6% BOG, 35.9% CWB), production of ornamental fish (P>0.05; BOG 42.3%, CWB 33.9%), and fish in aquarium exhibits (P>0.05; 37.9% BOG, 35.1% CWB) were perceived by respondents to cause moderate suffering.

### Perceptions about fish slaughter

**Fig 8:**
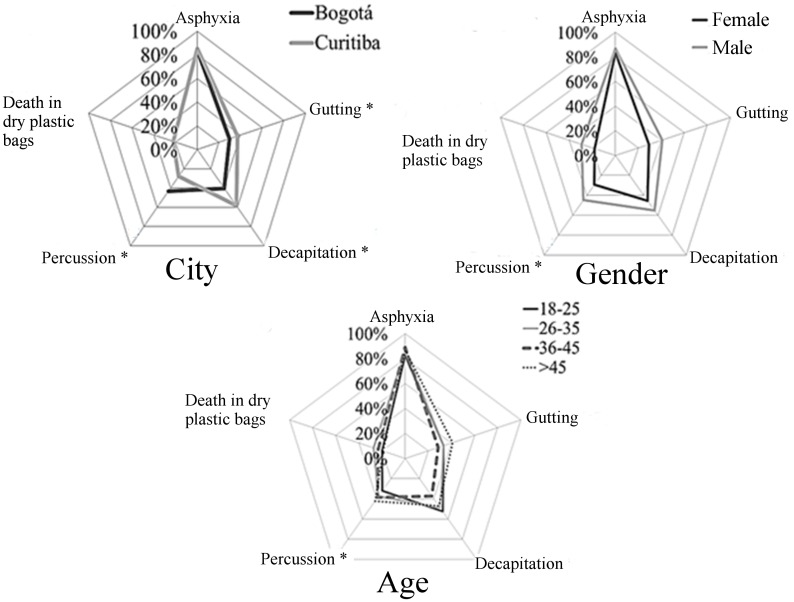
Percentage of responses regarding the slaughter methods (Q8) most observed by 782 citizens of Bogotá and Curitiba, 2014/2015. Data for city, gender and age, the asterisk indicates difference between groups (P<0.05; Chi-square test).

Results about respondent knowledge on slaughter methods are presented on Fig 8. Other methods mentioned by respondents from BOG were anesthesia (3.0%), exsanguination (1.3%) and electronarcosis (0.5%). Respondents from CWB also mentioned immersion in ice (4.5%) and electronarcosis (1.6%). Percussion and decapitation presented statistical difference between BOG and CWB (P<0.05; Fig 8). Besides that, answers about percussion differed according to gender and age (Fig 8). Men mentioned percussion method more than women did.

**Fig 9:**
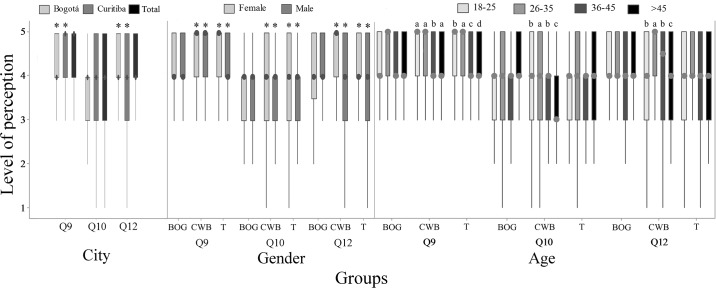
Perception of suffering in slaughter (Q9), meat quality (Q10) and regulation (Q12) by 782 citizens of Bogotá and Curitiba, 2014/2015. Data for city, gender and age; 1 = certainly not and 5 = certainly yes; the asterisk indicates gender difference (P<0.05, Mann-Whitney); letters indicate differences between age groups (P<0.05, Kruskal-Wallis and Dunn Test).

Most respondents (84.2% BOG; 90.4% CWB; P = 0.0027) believed that slaughter methods indicated on question Q8 (Table 1) may cause fish suffering (Fig 9). Higher scores for fish suffering were given by women and young groups (Fig 9, P<0.05). A total of 64.6% of respondents in BOG and 63.9% in CWB agreed that fish suffering may affect meat quality (P<0.05; Fig 9). Higher scores were observed on women and young groups in CWB (P<0.05; Fig 9).

Considering humane slaughter, 57.0% of respondents from BOG and 55.0% from CWB had never heard about it (P>0.05). In CWB, women were more familiar with the term humane slaughter (P = 0.009; 50.0%) when compared to men (36.4%). Additionally, only in CWB, the same was observed on young respondents of 18–25 years old (P = 0.005; 44.7%) when compared to age group older than 45 years old (23.4%). Most of respondents, 76.0% in BOG and 72.0% in CWB, agreed that fish should be included on humane slaughter regulation, with higher variation on CWB (P<0.05; Fig 9). Women and respondents under 45 years old presented higher scores than older respondents in CWB (Fig 9). Reasons given by respondents that agreed with the inclusion of fish on humane slaughter regulation were based on recognition of fish sentience, concerns about meat quality and equality among farm animals. For example, “As any other animal, fish are sensitive and feel pain. If fish are intended for human consumption, we should be careful to cause the least possible suffering”, woman from BOG, 26–35 and higher education; “to improve meat quality and increase added value, avoiding excessive suffering”, woman from BOG, 18–25 years old and higher education; “because, similarly to other animals, fish feel stress and pain”, woman from CWB, 18–25 years old and higher education; “less suffering and higher meat quality, following what happens on slaughter of broiler chickens, pigs and cattle in some slaughterhouses”, man from CWB, over 45 years old and higher education.

## Discussion

### Demographic data

The percentage of male and female respondents differed from gender distribution within the population of each city, that is 47.8% male and 52.2% female in BOG [31] and 47.8% male and 52.3% female in CWB [32]. A higher percentage of female respondents may be related to higher sensitivity and positive attitudes of women to animal protection [33]. Therefore, women may have greater motivation to complete such a survey.

Regarding education, a total of 92.9% of respondents from BOG and 97.5% from CWB were attending or attended higher education, differing from the population average of 22.1% in BOG [31] and 43.6% in CWB [32], which is most likely a consequence of the sampling method of our study that included most contacts from universities. The sampling method also explains the most prevalent age range of respondents. Additionally, significant difference in the number of respondents with secondary education between BOG and CWB (Table 2) seems unlikely to have much impact on survey results due to the low percentage of respondents within this education level.

### Fish sentience

#### Sentience perception according to geographic origin

The fact that most respondents considered fish as sentient beings, regardless of geographic origin, may be related to increased support for promoting changes in fish handling, aiming to prevent animal suffering. A small percentage of respondents presented a view that fish are unable to feel emotions, which conflicts with several studies that have reported behavioural complexity of fish, including data on cognition and consciousness [5–8,34]. Additionally, recently it was concluded that fish are able to retain memories of events with positive and negative valence, which may be retrieved by environmental cues [35]. Thus, fish behavioural complexity and flexibility evidence their sentience.

#### Sentience perception according to gender and age of respondent

Higher scores given to fish sentience by women are in accordance with other studies involving perception and attitudes towards animals, probably due to a higher sensitivity of women [33,36,37]. Regarding the effect of age and sentience perception in respondents from CWB, Kendall et al. [38] also reported a negative correlation between age and positive human attitudes towards the treatment of animals. Recent concerns about farm animal welfare in Brazil may also have influenced opinion, especially of younger citizens. However, reasons for the absence of significant differences among age groups in BOG are unknown; this result is in accordance with Signal and Taylor [39], who did not observe correlation between age and positive attitudes to animals. Citizens of CWB may be going through a generation shift towards higher recognition of sentience by the younger age groups, represented by greater variance of scores. The influence of age on perception of fish sentience should be further studied in both cities, taking into considerations personal beliefs, values and familiarity with animal production, as proposed by Vanhonacker and Verbeke [40].

#### Sentience perception according to animal species

Results suggest that respondents had more difficulty to score sentience in animals that are more phylogenetically distant from humans. Animals farther apart from humans, such as fish and invertebrates, were perceived to have a lower degree of sentience, and this inverse relation between phylogenetic and animal sentience was also mentioned by other authors [5,37,41]. Additionally, the difficulty of human beings to understand emotions in fish due to differences in body structure, facial expressions and living environment, may contribute to the attribution of low sentience levels in fish when compared to other animals [20]. Again, there was agreement between our results and the literature in the higher sentience scores given by women in all groups of animals [33,36,37], and by young people [38].

### Fish suffering

#### Perceptions about fishing

Catch-and-release angling is questionable from an ethical point of view because fish may be subjected to unnecessary suffering. This fact was recognized by some of our respondents. Ferguson and Tufts observed that removing fish from water for one minute after exhaustive exercise increased fish mortality when compared to a control group [42]. Cooke and Philipp also showed that the practice of capture and release increases predation risk for these animals [43]. Furthermore, removing fish from water has already been showed to be a stressing condition, with increased blood cortisol levels [8,44]. The recreational angling, with or without releasing, affects directly the animal as it causes injury associated with pain [8,45]. Additionally, the recreational angling with release is associated with a mortality rate of 30–68% which depends on the type of hook, fish size, location and number of perforations [45]. In general, the majority of fish are hooked through the jaw region [45]. This area is important for respiration, food acquisition and consumption, and in some cases for reproduction (e.g., mouth brooding, competition for mates) or social interactions (e.g., yawning, displays) [46]. Additionally, Sneddon reported several types of sensory receptors in this area, related to perception of pain [47]. As suffering is defined like an unpleasant state of mind that disrupts the quality of life, it is the mental state associated with unpleasant experiences such as pain, distress and injury. Thus, it seems that unnecessary suffering and poor welfare are consequences of catch-and-release angling in fish.

In the literature, level of support for catch-and-release angling varied in different countries, being 35.0% in Germany, 65.0% in New Zealand, 71.0% in England [26,48]. Respondents from BOG and CWB showed higher sensibility for fish suffering in catch-and-release angling, and the overall higher percentage of female respondents may have corroborated to this result.

In Brazil, fishing was regulated by the Ministry of Fisheries and Aquaculture, which was incorporated in October 2015 by the Ministry of Agriculture. Amateur fishing license is issued by governmental body to a person or an entity for leisure and sports when there are no economic purposes [49]. This license is in accordance to the article 29 of the Environmental Crimes Act, law 9,605, which considers crime the act of killing, pursuing, hunting, catching and using wild, native or migratory animals without specific license or in disagreement with the license [50]. Nevertheless, this is incoherent with article 32 of the same law, which considers a crime to practice acts of abuse, mistreatment, to injury or to mutilate wild or domestic, native or exotic animals. In Colombia, sport fishing license is issued to any person [51]. The same incoherence was observed in Colombian animal protection law, which considers cruelty to cause injury on animals [52].

Some groups have suggested the development of a code of practices to improve the welfare of fish on recreational fishing [48,53]. In 2007, the European Inland Fisheries Advisory Commission of the Food and Agricultural Organization of the United Nations prepared a code of practice for recreational fisheries, to set minimum global requirements for this practice, including fish welfare [54]. Codes of practice are non-mandatory, and the voluntary adoption by people involved with recreational fishing may be strongly related to personal belief about fish sentience. Additionally, as most respondents of both BOG and CWB do not agree with catch-and-release angling, it seems important to develop specific regulation to avoid unnecessary suffering of fish that is commonly observed in this practice.

We observed significant differences in reports regarding the practice of angling. Variation was also found when considering results in New Zealand (15.1%) [26] and Finland (40.0%) [55]. The variation may be consequence of cultural characteristics of each country. For example, in the metropolitan region of Curitiba there are fish-and-pay ponds for recreational fishing. Again the gender difference observed is in accordance with the literature. As observed by Shrestha et al., 99.0% of sport fishing in Pantanal, Brazil, were practiced by men [56]. These results may be related to higher concerns of women regarding fish welfare and sentience, leading to reduced enjoyment and practice of angling.

#### Perception of fish pain

Low percentages of respondents who do not believe fish feel pain were also found by Muir et al., in New Zealand [26], with 1.2% of total respondents. Scientific debates have pointed against [17,57] and in favor [6,7,58–61] of fish capacity to feel pain. However, the precautionary principle is recommended in order to improve animal welfare, which means that one should protect a group of animals if there are doubts about their sentience [59]. In addition, the perception of great part of respondents that fish can feel pain is important to encourage the adoption of procedures and the development of regulation to avoid fish suffering. This perception is supported by the majority of scientific papers on fish abilities to feel pain [6,7,58–61].

#### Perception of fish suffering in different scenarios

Vanhonacker et al found that attitudes, perceptions, concerns and behaviour were much more strongly influenced by variables related to individual experiences and familiarity with the agricultural sector and to life-style, values and beliefs in relation to animals, animal welfare and animal production than by socio-demographic characteristics [40]. Thus, the variance observed between cities regarding the perception of fish suffering in different scenarios may be related to individual experience of respondents that may affect suffering perception. More research is desirable to better understand the effect of age on perception of fish suffering in different scenarios.

The live fish fair, which was a scenario with high perception of animal suffering, has taken place for more than 25 years in metropolitan region of Curitiba during the Easter week. This may have affected suffering perception of older respondents, which presented lower scores in this scenario within the age groups (Fig 7). It is also common in other States in Brazil, such as Santa Catarina, Rio Grande do Sul, Paraíba and Pernambuco [28,62–65]. Live fish fair is an income complement for producers because it is the opportunity to sell the product directly to final consumer, without intermediaries. In Araucária, metropolitan region of CWB, for example, about 12,000 people visited the fair in two days [66]. High number of visitants seems to be related to a demand for fresh product. Most common species are tilapia (*Oreochromis niloticus*), common carp (*Cyprinus carpio*), grass carp (*Ctenopharyngodon idella*), big head carp (*Aristichthys nobilis*), silver carp (*Hypoththalmichthys molitrix*) and native species like catfish (*Rhamdia quelen*) and pacu (*Piaractus mesopotamicus*) [62].

During the live fish fair in Araucária, 20 tons of fish were sold in 2015 [67], and in Santa Maria, State of Rio Grande do Sul, amount sold reached 40 tons [62]. Usually, there is a fast period of 24 to 48 hours prior the fair. Fish are kept in tanks with high densities on farm, during transport and at the fair. Transport of live fish is considered a limitation by producers since transport requires time and increase production cost [65]. Fish are sold alive and consumers take them home in plastic bags without water supply; or fish are slaughetered and cleaned on place without previous stunning. Both situations are considered critical points for animal welfare [8,23,28,68], and similar slaughter situations with any other vertebrate farm animal would probably not be allowed by governamental bodies.

Based on high perception of fish suffering, it seems relevant to assess fish welfare during the fair to implement measures that may improve animal welfare or may suggest the need of abolishing live fish fair. Establishing a fish producer association to centralize fish slaughter and processing, supported by local authorities, may be a viable alternative to replace those fairs [65]. In this case, fresh fish would still be offered to consumers, avoiding unecessary suffering caused by poor welfare conditions on tanks during the fair and by asfixia in plastic bags.

The difference in the perception of fish suffering between the fish-and-pay and the fishing with hook and line scenarios is worrisome, since they have similar characteristics. On fish-and-pay pond establishments, consumers have the option to take home the cleaned fish, which may lead to a reduced perception of suffering, probably due to dissociation of the live animal from the final product that is consumed [69].

It is relevant that respondents showed perception of fish suffering in laboratories. The amount of fish used for research purposes has increased since the 1990s [70], and it tends to further increase. As an example, in the United Kingdom, the number of fish used in research increased by 15% from 2010 to 2011 [71]. Since fish represent the most diverse group of vertebrates, with aproximately 32,000 species [12], the development of guides for the care and use of these animals in research has been a challenge [70]. Other studies have observed opposition to the use of animal in research [41,72,73], which may indicate the need of further information about animal welfare in laboratory scenario. Welfare assessment of fish in laboratories is also desirable to identify critical points and provide actions to address them.

Results suggest that respondents may be aware of some items related to fish farming, fish in pet sore, production of ornamental fish and fish in aquarium exhibit scenarios to consider them as moderate suffering. In Brazil, 61.8% (765,287 tons) of fish for human consumption are obtained by capture and 38.2% (473,429 tons) by inland aquaculture [74]. In Colombia, these percentages are 40.0% (59,639 tons) and 60.0% (89,398 tons), respectively [74]. Procedures of fish handling, removal from the water, transport, disease control, stocking density, feed withdrawal and slaughter are known as critical points that lead to poor welfare in a great number of fish [68]. Public perception of animal suffering should be taken into consideration by governmental bodies to invest in research aiming to provide support for the development of regulation for the protection of fish.

Considering the pet store scenario, animal abuse in pet stores located in metropolitan region of Curitiba have been reported [75]. Although complaints did not include fish, they suggest inability of some pet stores to comply with minimum animal welfare requirements, demanding further investigation. According to Huntingford et al. and Volpato, potential critical points of fish kept in pet stores include high stocking density, low water quality and abnormal behavior, which refer to poor welfare [8,76]. In Brazil and Colombia, pet stores must contract a veterinarian, who is responsible for animal welfare [77,78]. Moderate perception of fish suffering in pet stores may indicate the need of more active participation of veterinarians on these establishments.

Perception of fish suffering in ornamental scenario may be influenced by previous knowledge of respondents about this production chain. According to FAO, more than 90% of freshwater ornamental fish are captive bred compared to only 0.3% in the case of marine fish [79]. Fish exported from Brazil are almost all captured in both continental and marine waters, while internal market is mainly supplied by inland captive fish [80]. Thus, perception of moderate suffering may be related to those fish obtained from aquaculture, which may be understood by respondents as having similar conditions of fish bred for human consumption. However, animal suffering may be even higher in captured wild fish, since it is estimated that only one in ten animals captured from nature survives [81]. Although the estimate does not include fish, high mortality is also expected to occur. Great losses of wild-caught fish occur because fish are subjected to physical injury, extreme changes in water quality and temperature, and exposure to chemicals used as prophylactic treatment for disease control [82]. Our results suggest the need of more information to consumer about the origin and keeping of ornamental fish, as well about welfare status of these animals.

Moderate perception of suffering in fish in aquarium exhibits may be related to conditions of aquariums, where animal welfare status is controversial [8,76,83]. Respondents perceived fish kept as pets as a low suffering scenario (37.9% BOG; 35.1% CWB), which may be motivated by a common sense that pet owners care about their pets. In the specific case of fish, water quality, inadequate nutrition and privation of social interaction are critical points to animal welfare [8].

Perception of fish suffering in different scenarios may motivate new studies about animal welfare assessment. From such knowledge, establishments may implement protocols to ensure minimum standards of animal welfare. An effective communication on the level of fish welfare in different scenarios can value the work of responsible professionals and improve public perception.

### Perceptions about fish slaughter

The variation in percentages of citation of different slaughter methods may be a consequence of previous contact of respondents with them. Additionally, the fact that men mentioned percussion more often than women did may be explained because percussion stunning is likely the most common method used by anglers when fish is killed for harvest [46] and, according to our results, angling is predominantly practiced by men. The perception of fish suffering in different slaughter methods is in agreement with Pedrazzani et al., who observed percentages of 85.0% and 89.0% [28]. Slaughter methods mentioned on question Q8 are known to cause animal suffering and are not recommended [84]. The development of new methods of fish slaughter is in agreement with advances of society’s ethical concerns and with new demands of fish welfare of the Word Organization for Animal Health–OIE [18].

Pedrazzani et al. observed lower percentages of 52.9% and 44.1% from respondents who believed fish suffering would affect meat quality [28]. High education level of our respondents may have contributed to higher knowledge about the relation between animal suffering and meat quality. Procedures such as pre-slaughter, catching and transport may cause fish stress [84], and there is a negative correlation between level of suffering and meat quality [85]. Since expected quality is one of the most important factors in consumer intention to purchase food [86], it seems important to inform consumers about the relationship between animal suffering and meat quality in order to increase demand for higher welfare on fish production for human consumption.

The difference between percentage of respondents who never heard about humane slaughter in our results and that of Pedrazzani et al. [28], who observed higher percentage of 91.1%, may be a consequence of higher educational level of respondents in our research. Therefore, on general population of BOG and CWB, where educational level is lower [31,32], percentage of people who does not know about humane slaughter is expected to be even higher. Many people enjoy eating meat but few enjoy harming or killing other sentient creatures. These inconsistent beliefs create a ‘‘meat paradox”; people simultaneously dislike hurting animals and like eating meat [87]. To solve this problem, people become vegetarian or fail to recognize that animals are killed to produce meat. Although few people live in true ignorance, some meat-eaters may live in a state of tacit denial, failing to equate beef with cow or pork with pig [87]. Perhaps for this reason, respondents do not know the meaning of humane slaughter. Society awareness about the existence of procedures of fish slaughter that prevent animals from feeling pain is an important tool to increase demand for stunning methods on fish production. This is particularly important because in Colombia [25] and in Brazil [24], regulations about humane slaughter of farmed animals do not include fish.

There was a positive attitude of participants towards the inclusion of fish on the existing regulation in both countries, with higher scores in BOG. Besides the fact that half of respondents did not know the term “humane slaughter”, results suggest that they understood its meaning and expressed their opinion.

## Conclusions

This study presents original data suggesting that highly educated citizens from Bogotá and Curitiba consider fish sentient beings. There was less variability in responses from citizens in BOG as compared to those in CWB; the latter showing slightly lower scores of sentience and suffering perception, apparently due to responses from higher age-groups. The perception of suffering in specific scenarios challenges activities such as recreational fishing, live fish fair and laboratory fish use. There seems to be a need to better inform citizens about procedures applied to fish in other scenarios, where suffering was not considered high by respondents, such as ornamental fish, aquarium exhibits and fish kept as pets. Recognition of fish suffering also endorses humane slaughter regulations to reduce pain in a large number of fish slaughtered annually for human consumption in Colombia and Brazil. Overall, our results suggest that well educated citizens from Bogotá and Curitiba may accept the inclusion of fish in the circle of moral consideration and also suggest the need to improve communication between science and society. It remains important to study public attitude in other regions of Latin America, since cultures, even within the same country, may be related to different perceptions.

## Supporting Information

S1 Dataset Answers in the original language of citizens from Bogotá and Curitiba. (XLSX)
